# Supplementary material for: In-silico study of accuracy and precision of left-ventricular strain quantification from 3D tagged MRI
Source: PLoS One. 2021 Nov 5;16(11):e0258965. doi: 10.1371/journal.pone.0258965 (PMC8570486; doi:10.1371/journal.pone.0258965)
Supplement: S1 File — (DOCX) [file pone.0258965.s001.docx]

S1 Fig represents the dependency of the optimal regularization parameter on image resolution and SNR. The color code shows the value of regularization parameter ranging from 0.0 to 0.3. For isotropic image resolution (S1 Fig.a), change in SNR does not affect the value of optimal regularization strength for small pixel size (1 mm). However, for larger pixel size, regularization strength increases with decreasing SNR. Similarly, for a given SNR, increasing pixel size is compensated with higher regularization strength. For the worst case (pixel size = 3.5 mm and SNR 5), the image registration requires *β* = 0.2. For anisotropic image resolution (S1 Fig.b), the dependency of regularization strength on SNR and pixel size follows the same trend as in isotropic images. For the worst case (pixel size = 3.5x7.0x7.0 mm^3^ and SNR 5), *β* = 0.3 is required.

**
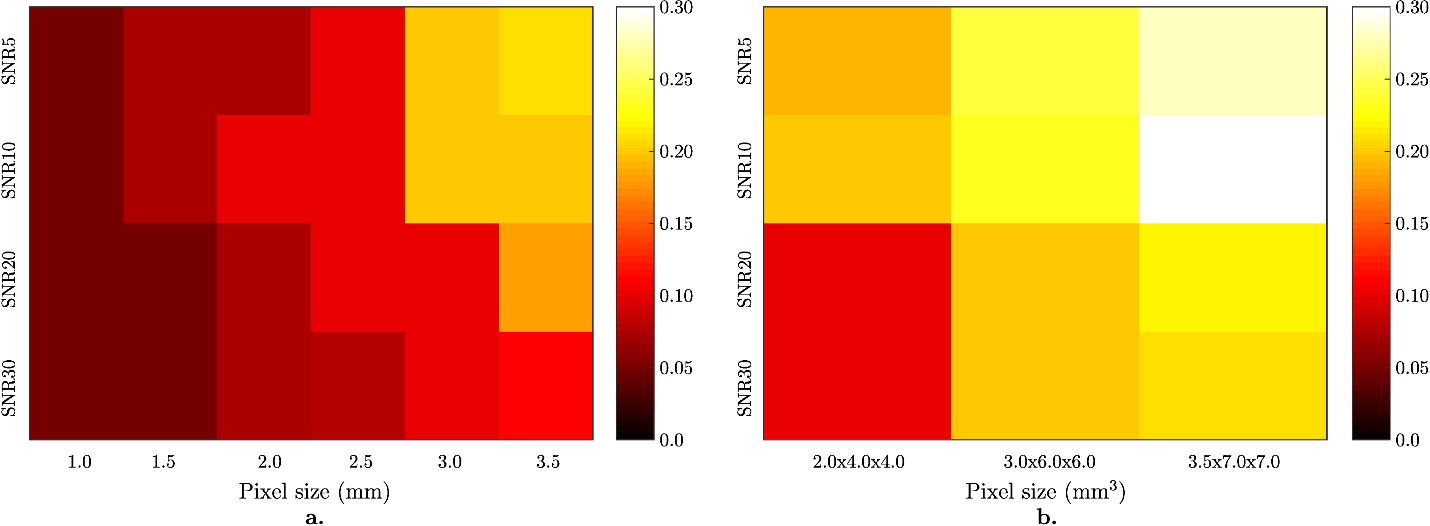
**

**S1 Fig. Change in optimal regularization strength with respect to image resolution and SNR.** Colour code represents the value of regularization strength. The pixel size (in mm) in one direction is shown on x axis for isotropic images (a). SNR has more impact on the choice of regularization strength as pixel size increases. For anisotropic images, 3D image resolution is represented on x axis (in mm^3^) (b). For a given SNR, increasing pixel size requires more regularization. Likewise, higher regularization is required for decreasing SNR when pixel size is kept constant.

The regional changes in normalized absolute displacement error (%) and mean ± standard deviation in strain component errors are plotted for IP and TP shifts in S2 Fig and S3 Fig, respectively. For any kind of shift, we expect the maximum error to occur in the direction of the shift, where the tag lines are blurred. Hence, for IP shift (S2 Fig), the errors are plotted with respect to a change in in-plane angle, *α*, where the shift is applied at *α* = 0°, *α* = 180° and *α* = 360°, shown with the green arrows on S2 Fig.b and S2 Fig.c. For both the displacement (S2 Fig.a) and strain components (S2 Fig.b, c), maximum errors are observed for the largest amount of shift, ±6 mm (shown by the red curves). When the image stacks are shifted in-plane, the smallest error is found in opposing sectors orthogonal to the direction of the shift. Hence, we observe some kind of periodicity in the distribution of radial (S2 Fig.b) and circumferential (S2 Fig.c) strain errors as a function of sector position, while the longitudinal component (S2 Fig.d) remains insensitive.

The effect of TP shift (S3 Fig), however, is better observed in the longitudinal direction. For TP shift, we expect to see more error towards base and apex where the tag information is lost due to shifting in the longitudinal direction. For the maximum amount of shift (±6 mm), the displacement and radial strain error get larger towards the apex and base compared to lower amounts of shift. Circumferential (S3 Fig.c) and longitudinal (S3 Fig.d) strain errors are less sensitive to TP shift.

**
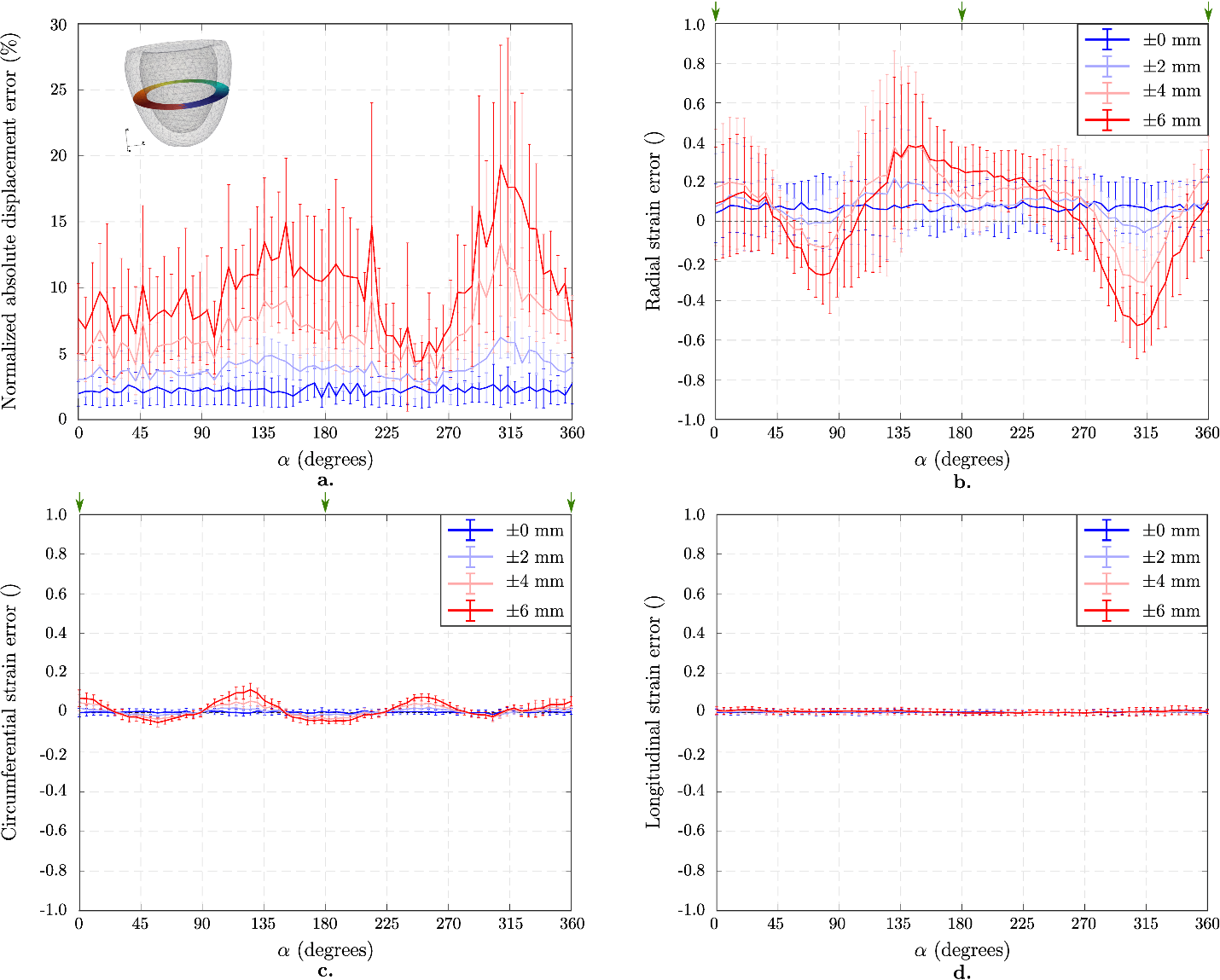
**

**S2 Fig. Effect of in-plane shift on regional motion quantification.** Results on images with anisotropic pixel size 3.5x7.0x7.0 mm^3^ and SNR 20 for 7 mm tag distance. For all the plots, colour code shows the amount of in-plane shift ranging from ±0 mm to ±6 mm. Mean ± standard deviation in normalized displacement error norm (%) plotted as a function of in-plane angle, *α* with respect to shift direction (a). Mean ± standard deviations in component-wise Green-Lagrange strain error as a function of in-plane angle (b-d). Increasing amount of shift leads to an increased error in displacement field (a). Radial and circumferential strain errors show the dependency on in-plane angle, especially for the maximum amount of shift, ±6 mm. The shifts are applied at *α* = 0°, *α* = 180° and *α* = 360°, shown with the green arrows on top (b, c). Longitudinal component is insensitive to any shift (d).

**
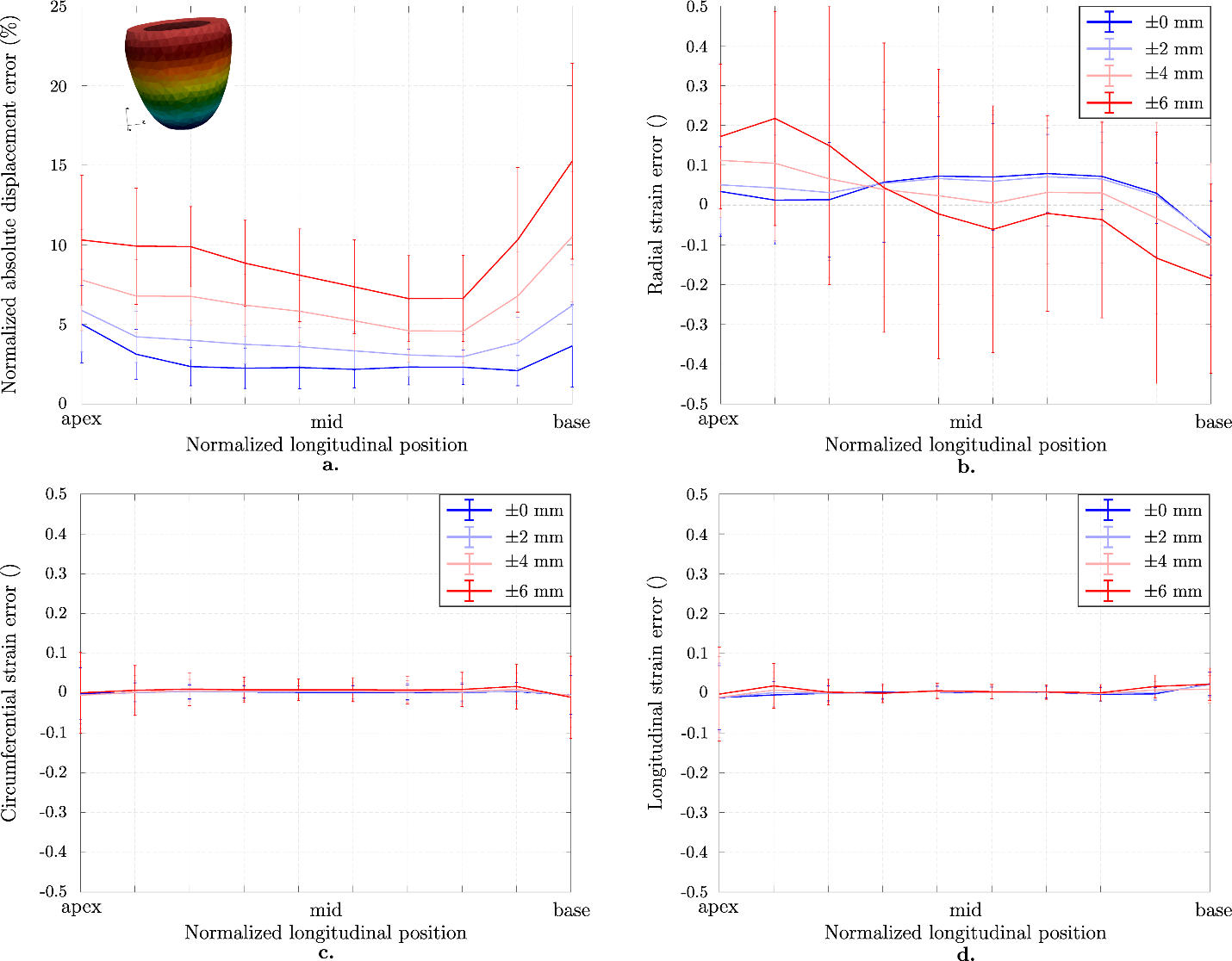
**

**S3 Fig. Effect of through-plane shift on regional motion quantification.** Results on images with anisotropic pixel size 3.5x7.0x7.0 mm^3^ and SNR 20 for 7 mm tag distance. For all the plots, colour code shows the amount through-plane shift ranging from ±0 mm to ±6 mm. Mean ± standard deviation in normalized displacement error (%) plotted as a function of normalized longitudinal position (a). Mean ± standard deviations in component-wise Green-Lagrange strain error as a function of normalized longitudinal position (b-d). Increasing amount of shift leads to an increased error in displacement field (a). Radial strain error increases towards the base and apex, especially for the maximum amount of shift, ±6 mm, while longitudinal and circumferential components are insensitive to any shift (d).
