# Supplementary material for: In-silico study of accuracy and precision of left-ventricular strain quantification from 3D tagged MRI
Source: PLoS One. 2021 Nov 5;16(11):e0258965. doi: 10.1371/journal.pone.0258965 (PMC8570486; doi:10.1371/journal.pone.0258965)
Supplement: Attachment — Submitted filename: Response to Reviewers.pdf [file pone.0258965.s002.pdf]

We would like to thank both reviewers for carefully reading our manuscript, and making constructive comments. We have addressed all reviewers' comments in the following point by point response:

Reviewer #2

The present manuscript evaluates a finite-element method for strain quantization using an in-silico phantom. The approach of using an in-silico phantom is likely of interest to investigators in the field and the analysis of how parameters like tag distance and pixel size affect the accuracy of the strain quantification is important. The manuscript does not open completely new ground in the field, but it could make a useful contribution to the field, and the results are important for expanding our current knowledge about the limits of strain quantification with current MRI methods and post-processing techniques. This comment is also not meant to the play down the indisputable technical sophistication of the work.

Thank you very much.

The manuscript could be improved by avoiding some errors like the use of terms or abbreviations before they are properly defined, providing some more information about the in-silico phantom, and in general helping the reader gain an easier intuitive understanding of the results and the fundamental limits to reducing strain error.

We have carefully revised the manuscript to define abbreviations at their first occurrence.

Regarding the data sharing policy of PLOS journals, we would like to state that we have no restrictions to share the analysis data for this study. The minimal dataset is publicly available at ETH Library, https://www.research-collection.ethz.ch/handle/20.500.11850/497459 and a DOI will be provided after the institutional approval. The MATLAB scripts required to generate all the datasets used in this study are publicly available (https://gitlab.inria.fr/mgenet/dolfin_warp).

1) In-silico phantom: it would be useful to have some basic information about the phantom in terms of ES and ED cavity volume, mean wall thickness at ED and ES.

In this study, we used a generic anatomical model of the LV with a simple geometry represented by a truncated ellipsoid. The phantom has an ED volume of 202 ml, which decreases down to 62 ml at ES. The ventricular wall thickness is almost constant across the ventricle with an approximate ED mean value of 15 mm, which increases up to approximately 22 mm at ES. The tags are introduced at ED in the synthetic images, consequently there are between ca. 2 (for 7 mm tagging) and 5 (for 3 mm tagging) tag lines within the thickness of the myocardium. This is now stated in Section 1.1:

In this study, we utilize a generic anatomical model of the LV represented by a truncated ellipsoid with an approximate end-diastolic volume of 202 ml, decreasing down to 62 ml at end-systole (ES). The ventricular wall thickness is almost constant across the ventricle with approximate values of 15 mm and 22 mm at end-diastole (ED) and ES, respectively.

And Section 1.2:

The three orthogonally tagged image stacks are combined for each time frame according to:

$$I = \sin\left(\frac{\pi \boldsymbol{X}_i}{s}\right), \tag{1}$$

where $\mathbf{X}_i$ corresponds to the spatial coordinate of the image voxel in the ith direction, and s is the tagging distance, ranging from 3 mm and 7 mm (Fig 2.a). Considering the approximate ventricular wall thickness at ED, the synthetic images have between ca. 2 (for 7 mm tagging) and 5 (for 3 mm tagging) tag lines within the thickness of the myocardium.

In particular the relation between mean wall thickness and tag-line spacing is of some relevance for understanding when increased tag-line distance impacts on the accuracy of radial strain estimation. In fact, in the case of radial strains it may be useful to not only assess strain quantification as a function of TPR but also as a function of the tag-line-distance to mean wall-thickness ratio. It should be made clearer to the reader that for radial strain quantification it is important that at least two intersection points/lines fall within the wall thickness when moving in the radial direction. This limitation is much less of a constraint for the circumferential and longitudinal components.

We agree with the reviewer that TPR is not the only dimensionless parameter that is important for strain quantification, and that wall thickness to tag distance is also at play. Additionally, a characteristic length of the kinematics with respect to the tag distance would be of interest. However, in order to limit the scope of the study—which is already quite large—and to prevent confusion, we decided to fix the biomechanical model parameters (both geometrical and material, which control the kinematics) to normal values, and only study the impact of imaging parameters. We clarified this very relevant point in Section 3:

The elevated error in the radial component can also be associated to the thickness of the myocardial wall, which is much smaller than the circumference and length of the ventricle [44]. However, in this study, we did not investigate the effect of wall thickness on motion quantification in detail. Instead, we limited the scope of the study to the effect of imaging parameters only, fixing the geometrical and material biomechanical model parameters to normal values.

We also clarified the point regarding the need for balanced tag distances (large enough to prevent tag jumping during tracking; but small enough to maintain contrast) in Section 3:

Therefore, we studied the effect of TPR in the optimal range of tag distance; coarse enough to prevent the tag line jumping during tracking and fine enough to have good image contrast.

2) Page 4: it may be better to use different symbols for the functional J in equation 6 and the quantity J in equation 7, as they apparently represent different things.

We thank the reviewer for the correction. The same variable, J, was mistakenly used to represent the cost function and $\det \mathbf{F}$ in Equation 4 and Equation 7, respectively. We have revised the equation as shown below. J in Equation 4 has been changed to variable h to represent the cost function while keeping $\det \mathbf{F}$ still represented by J in Equation 7.

$$\text{find } \mathbf{U} = \underset{\{\boldsymbol{U}\}}{\text{argmin}} = \left\{h(\boldsymbol{U}) = (1-\beta)\Psi^{\text{im}}(\boldsymbol{U}) + \beta\Psi^{\text{reg}}(\boldsymbol{U})\right\}, \quad (4)$$

3) Equation 4: Generally, with regularization the primary quantity that is to be minimized - in this case the similarity metric - is not weighted by the regularization parameter. Why did the authors choose in this case to use a (1-beta) factor to weigh the similarity metric, in addition to the customary weighing of the regularization term by beta? Is there any logic behind requiring that the two weighting factors to sum to one, i.e. choosing weighting factors of (1-beta) and beta?

We thank the reviewer for this comment. The reason why we use (1-beta) and (beta) to weight the similarity metric and regularization energy, respectively, is to have a convenient way of handling the range of regularization strength. It is just a way to rescale the regularization strength such that it belongs to $[0,1]$, not $[0,\infty]$. We briefly explain the weighting strategy in Section 1.3:

Hence, the problem of finding the displacement field can be formulated as

$$\text{find } \mathbf{U} = \underset{\{\boldsymbol{U}\}}{\text{argmin}} = \left\{h(\boldsymbol{U}) = (1-\beta)\Psi^{\text{im}}(\boldsymbol{U}) + \beta\Psi^{\text{reg}}(\boldsymbol{U})\right\}, \quad (4)$$

which aims to minimize the functional h expressed in terms of regularization strength β. The image similarity metric, Ψ^{im}, and the regularization energy, Ψ^{reg}, defined below, are weighted by factors (1- β) and β, respectively, to rescale the regularization strength to stay in the range $[0,1]$. Image similarity metric

$$\Psi^{\text{im}}(\mathbf{U}) = \frac{1}{2}\|I_t \circ \boldsymbol{\Phi} - I_0\|^2_{L^2(\Omega_0)}, \quad (5)$$

and regularization energy

$$\Psi^{\text{reg}} = \sum_K \frac{1}{2}\|\mathbf{Div}(\mathbf{F}.\mathbf{S})\|^2_{L^2(K)} + \sum_F \frac{1}{2h}[\![\mathbf{F}.\mathbf{S}.\mathbf{N}]\!]^2_{L^2(F)} \quad (6)$$

where **K**, **F** and **N** represent the set of finite elements, the set of interior facets, and the facets normal, respectively.

4) Page 4, choice of regularization parameter value: for practical application of the proposed method the regularization parameter would have to be chosen without recourse to the ground truth, which in practice means using some heuristic like the L-curve criterion or similar. Could the authors please comment on this and also relate this to the question why they weighed both terms in equation 6 by (1-beta) and beta, respectively.

As mentioned above, it is convenient to have a predefined range for the regularization strength to facilitate the investigation of range for beta in which the tracking algorithm performs well. For a more detailed discussion on this, the reviewer is referred to [1], where a range of regularization strength is examined for the regularization technique used in this study in case the ground truth is not known. It was shown that one can specify a range of beta where regularization has only minor effect on the results, to this end, it is possible to take a canonical value for all cases. On contrary for the case of hyperelastic regularization, however, it is not feasible to define an optimal value for beta. We have clarified this point in Section 1.3:

In this study, for each image set, we run the registration algorithm for a range of regularization strength β and use the best performing value (i.e. estimated motion closest to ground truth) in the analysis. The reader is referred to [1] for a more detailed discussion on the choice of β in case the ground truth is unknown.

How does this affect the choice of optimal regularization parameter when the ground truth is not known?

It should be noted that this study does not aim to develop any automatic way of calibrating the regularization strength. We claim that designing this "optimal" registration technique provides us with the error due to image content itself, not the image processing approach.

In order to clarify the dependency of optimal regularization parameter on image resolution and SNR, we added S1 Fig to the Appendix. The color code shows the value of regularization parameter ranging from 0.0 to 0.3. For isotropic image resolution (S1 Fig.a), decrease in SNR does not affect the value of optimal regularization strength for fine image resolution (1 mm). However, image registration requires higher regularization as both the image resolution and SNR decrease. For the worst case (image resolution = 3.5 mm and SNR 5), the image registration requires beta = 0.2. For anisotropic image resolution (S1 Fig.b), we again see a similar trend; higher regularization strength for low image resolution and SNR. For the worst case (image resolution = 3.5x7.0x7.0 mm^3 and SNR 5), beta = 0.3 is required.

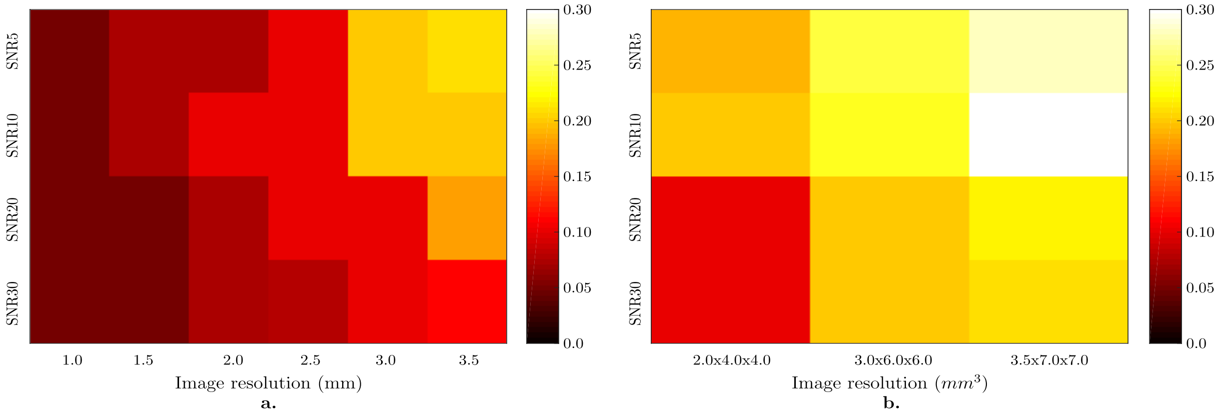

S1 Fig. Change in optimal regularization strength with respect to image resolution and SNR. Colour code represents the value of regularization strength. The image resolution (in mm) in one direction is shown on x axis for isotropic images (a). SNR has more impact on the choice of regularization strength as image resolution degrades. For anisotropic images, 3D image resolution is represented on x axis (in mm3) (b). The tracking algorithm requires a higher regularization strength as image resolution and SNR decrease.

Appendix

S1 Fig represents the dependency of the optimal regularization parameter on image resolution and SNR. The color code shows the value of regularization parameter ranging from 0.0 to 0.3. For isotropic image resolution (S1 Fig.a), a decrease in SNR does not affect the value of optimal regularization strength for small voxel size (1 mm). However, image registration requires higher regularization as both the image resolution and SNR decrease. For the worst case (image resolution = 3.5 mm and SNR 5), the image registration requires β = 0.2. For anisotropic image resolution (S1 Fig.b), we again see a similar trend; higher regularization strength for low image resolution and SNR. For the worst case (image resolution = 3.5x7.0x7.0 mm^3 and SNR 5), β = 0.3 is required.

We also referred to S1 Fig in the manuscript where we need to mention the optimal beta values; in Section 2.1.3:

The reader is referred to S1 Fig.a for the choice of optimal β values for varying image resolution and SNR.

And in Section 2.2.2:

The reader is referred to S1 Fig.b for the choice of optimal β values for anisotropic image resolutions (2.0x4.0x4.0 mm^3, 3.0x6.0x6.0 mm^3 and 3.5x7.0x7.0 mm^3) and varying SNR.

5) TPR (Tag distance to Pixel size Ratio) is first defined in Results, but the abbreviation is used beforehand in Methods. The authors should define new symbols or abbreviation at first use in the manuscript.

We apologize and have corrected this in the revised manuscript in Section 1.4:

In this study, we investigate both the individual and combined effects of two image characteristics, Tag distance to Pixel size Ratio (TPR) and SNR on images with isotropic (Fig 2) and anisotropic (Fig 3) spatial resolution.

6) The nature of the shifts that were applied to analyze the effects of varying degrees of geometric inconsistencies are not clear. In what direction where shifts applied to the images? Would a shift applied in one direction not result in different degrees of accuracy depending on the location of a myocardial segment relative to the center of the LV and the direction of the shift? This is rather confusing, and the authors need to make a better effort in explaining their method of evaluating geometric inconsistencies!

We thank the reviewer to help us clearing this point. In case no shift is present, three image stacks tagged in X, Y, and Z directions are aligned in space and combined to have the 3D tagged image. In this study, we simulated two types of shifts: in-plane (IP) and through-plane (TP), where the image stack Z is kept constant for both cases while the two other stacks are shifted to mimic inconsistencies during in-vivo imaging. Most 3D tagged studies are based on the work of Rutz et al. [2] in which line tagging with varying orientation is applied in three consecutive image volume acquisitions. Each of the line tagged imaging volumes is acquired in a single breath hold which is prone to volume misalignment due to inconsistencies in breath hold positions. We report the effect of shifting only globally, not sector-wise. Therefore, the shifting effect is mostly represented by the error bars, not the means.

In order to investigate the effect of shifting further, the regional changes in normalized absolute displacement error (%) and mean ± standard deviation in strain component errors are plotted for IP and TP shifts in S2 Fig and S3 Fig, respectively. In any kind of shift, we expect the maximum error to occur in the direction of the shift, where the tag lines are blurred. Hence, for IP shift (S2 Fig), the errors are plotted with respect to a change in in-plane angle α, where the shift is applied along the direction of α = 0° / 180°, indicated by the green arrows on S2 Fig.b and S2 Fig.c. For both the displacement (S2 Fig.a) and strain components (S2 Fig.b, c), maximum errors are observed at the maximum amount of shift, ±6 mm (shown by the red curves). When the image stacks are shifted in-plane, similar errors are found in the opposing sectors orthogonal to the direction of shift. Hence, we observe some kind of periodicity in the

distribution of radial (S2 Fig.b) and circumferential (S2 Fig.c) strain errors as function of sector position, while longitudinal component (S2 Fig.d) remains insensitive.

The effect of TP shift (S3 Fig), however, is better observed in the longitudinal direction. In TP shift, we expect to see more error towards base and apex where the tag information is lost due to shifting in longitudinal direction. For the maximum amount of shift (±6 mm), the displacement and radial strain error get larger towards apex and base compared to lower amounts of shift. Circumferential (S3 Fig.c) and longitudinal (S3 Fig.d) strain errors are less sensitive to TP shift.

In the revised manuscript, we explained how IP and TP shifts are applied in Section 1.4:

In this study, we simulated in-plane (IP) and through-plane (TP) shifts of image stacks with 3.5x7.0x7.0 mm^3 resolution and SNR 20. For this purpose, the image stack tagged in Z is kept constant for both cases while the two other stacks are shifted in the respective planes by the same amount (Fig 3).

Moreover, Fig 1 and Fig 2 are added as Appendix with the explanation below to show the localized effects of shifting. This is mentioned in Section 2.2.3:

The localized effects of shifting are further discussed in Supporting Information.

In order to illustrate the spatial mismatch better, Fig 3 has been refined using the ED configuration, and in accordance, Fig 2 was adjusted.

Appendix

The regional changes in normalized absolute displacement error (%) and mean ± standard deviation in strain component errors are plotted for IP and TP shifts in S2 Fig and S3 Fig, respectively. For any kind of shift, we expect the maximum error to occur in the direction of the shift, where the tag lines are blurred. Hence, for IP shift (S2 Fig), the errors are plotted with respect to a change in in-plane angle, α, where the shift is applied at $\alpha = 0°$, $\alpha = 180°$ and $\alpha = 360°$, shown with the green arrows on S2 Fig.b and S2 Fig.c. For both the displacement (S2 Fig.a) and strain components (S2 Fig.b, c), maximum errors are observed for the largest amount of shift, ±6 mm (shown by the red curves). When the image stacks are shifted in-plane, the smallest error is found in opposing sectors orthogonal to the direction of the shift. Hence, we observe some kind of periodicity in the distribution of radial (S2 Fig.b) and circumferential (S2 Fig.c) strain errors as a function of sector position, while the longitudinal component (S2 Fig.d) remains insensitive.

The effect of TP shift (S3 Fig), however, is better observed in the longitudinal direction. For TP shift, we expect to see more error towards base and apex where the tag information is lost due to shifting in the longitudinal direction. For the maximum amount of shift (±6 mm), the displacement and radial strain error get larger towards the apex and base compared to lower amounts of shift. Circumferential (S3 Fig.c) and longitudinal (S3 Fig.d) strain errors are less sensitive to TP shift.

7) Could the authors indicate in which cases (figures) the change of strain parameter or error is significant? It looks like in many cases the change of one parameter (e.g. SNR in the case of Figure 8) has little effect on the quantification error!

We thank the reviewer for this comment. Although we observe an increase in strain error when image resolution and SNR are decreased separately (Fig 4 and Fig 5, respectively), the combined effect of these two imaging parameters is more pronounced on the tracking error as shown in Fig 6 and Fig 8. For the lowest image resolution 3.5x7.0x7.0 mm^3 and SNR = 5, we observe the largest error in displacement field and radial strain. Ground truth displacements are utilized to derive the best estimate possible from the images to distinguish between the errors that could be induced by the image quality and the error due to tracking itself. Based on this, in Fig 8, although the error mean does not change significantly, we can see the effect of image quality on the error bars while the errors are quite low compared to in-vivo case.

We explained that in Section 3:

The effect of SNR is more pronounced for the estimation of the displacement field and radial strain when combined with the anisotropic voxel sizes (see Fig 7 and Fig 8). Although the anisotropic images represent a better approximation of the in-vivo imaging setting, we do not observe a dramatic increase in error for low SNR values by the choice of optimal β (Fig 8.a,b).

I think that indicating which changes are statistically significant is better than just speaking of "a considerable increase" of quantity X or similar.

We performed a two-way ANOVA analysis to determine the contribution of SNR and image resolution to the tracking error. For isotropic image registration (Fig 6), ANOVA analysis showed that the individual contributions of SNR and image resolution in normalized displacement error are 56% and 39%, respectively (for $p < 0.0001$). Similarly, SNR is the dominant source of variation for radial and circumferential strain components, with 69% and 39%, respectively, while the corresponding contributions by image resolution are 20% and 37%. For the longitudinal component, largest source of error is image resolution with 72% while it is 17% by SNR (for $p < 0.0001$). We have reported these values in Section 2.1.3:

The two-way ANOVA analysis on SNR and image resolution shows that SNR is the main source of error in displacement field, radial and circumferential strain errors with 56%, 69% and 39%, respectively, while the corresponding contributions from image resolution are 39%, 20% and 37%. The longitudinal strain error, however, is 17% due to SNR and 72% due to image resolution ($p < 0.0001$).

For the anisotropic image analysis (Fig 8), SNR is the main source of error in normalized absolute displacement and radial, circumferential and longitudinal strain errors with values 62 %, 78%, 64%, and 46%, respectively while image resolution contributes by 36%, 1%, 16%, and 24% to each field (for $p < 0.0001$). We have reported these values in Section 2.2.2:

ANOVA analysis shows that SNR is the main source of error in displacement and radial, circumferential and longitudinal strain errors with values of 62 %, 78%, 64%, and 46%, respectively while image resolution contributes with 36%, 1%, 16%, and 24% to each field (for $p < 0.0001$).

8) The figures are appended in the manuscript PDF in reverse order (in terms of the numbering).

We apologize for this mistake and have corrected the figure order in the re-submission.

9) Equation 1: The image-related quantity X_i is not explained.

We apologize for having missed the definition of $\mathbf{X}_\mathrm{i}$ in the manuscript. $\mathbf{X}_\mathrm{i}$ represents the spatial coordinate of the image voxel in the ith direction. Following correction has been made in the Section 1.2:

The three orthogonally tagged image stacks are combined for each time frame according to:

$$I = \sin\left(\frac{\pi \boldsymbol{X}_i}{s}\right), \tag{1}$$

where $\mathbf{X}_\mathrm{i}$ corresponds to the spatial coordinate of the image voxel in the ith direction, and s is the tagging distance, ranging from 3 mm and 7 mm (Fig 2.a). Considering the approximate ventricular wall thickness at ED, the synthetic images have between ca. 2 (for 7 mm tagging) and 5 (for 3 mm tagging) tag lines within the thickness of the myocardium.

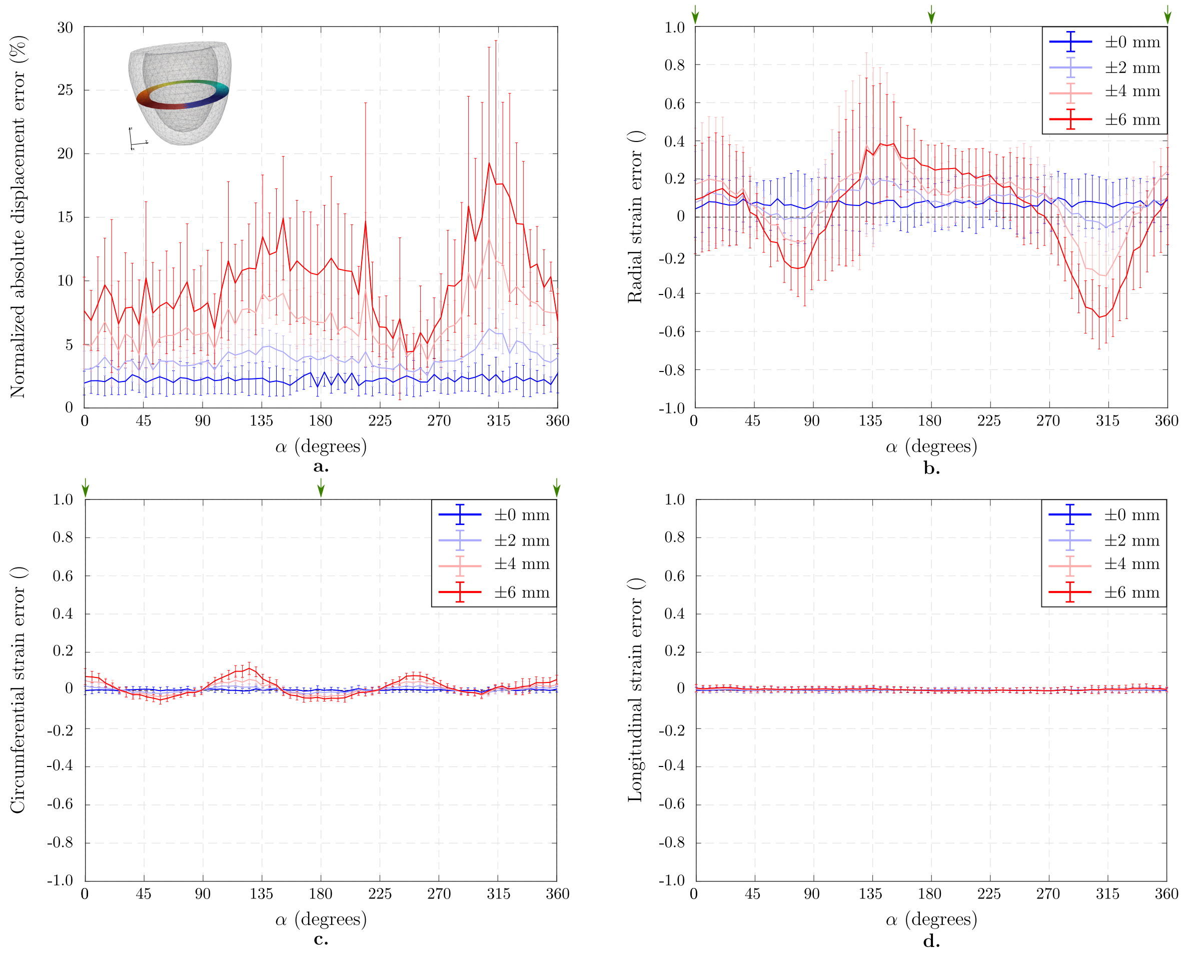

S2 Fig. Effect of in-plane shift on regional motion quantification. Results on images with anisotropic image resolution 3.5x7.0x7.0 mm3 and SNR 20 for 7 mm tag distance. For all the plots, colour code shows the amount of in-plane shift ranging from ±0 mm to ±6 mm. Mean ± standard deviation in normalized displacement error norm (%) plotted as a function of in-plane angle, α with respect to shift direction (a). Mean ± standard deviations in component-wise Green-Lagrange strain error as a function of in-plane angle (b-d). Increasing amount of shift leads to an increased error in displacement field (a). Radial and circumferential strain errors show the dependency on in-plane angle, especially for the maximum amount of shift, ±6 mm. The shifts are applied at α = 0°, α = 180° and α = 360°, shown with the green arrows on top (b, c). Longitudinal component is insensitive to any shift (d).

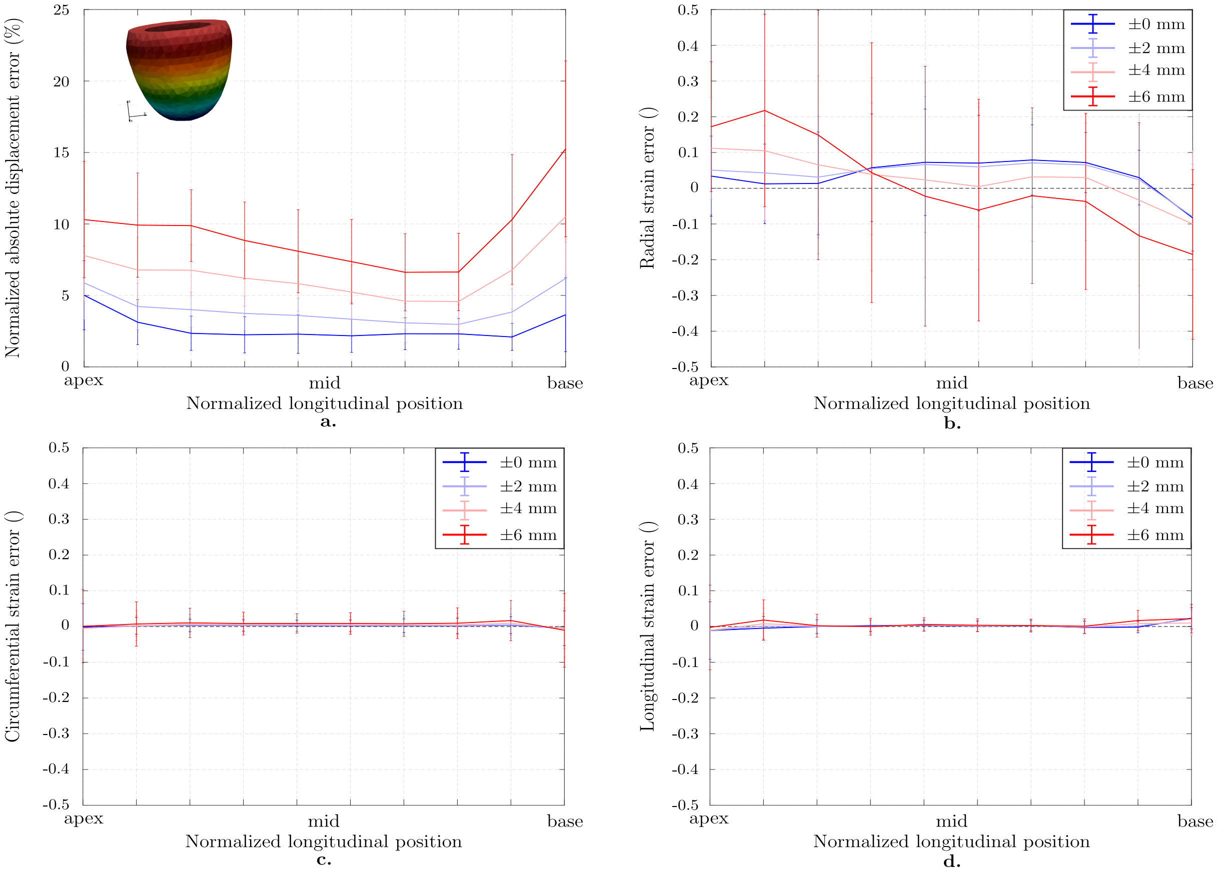

S3 Fig. Effect of through-plane shift on regional motion quantification. Results on images with anisotropic image resolution 3.5x7.0x7.0 mm3 and SNR 20 for 7 mm tag distance. For all the plots, colour code shows the amount through-plane shift ranging from ±0 mm to ±6 mm. Mean ± standard deviation in normalized displacement error (%) plotted as a function of normalized longitudinal position (a). Mean ± standard deviations in component-wise Green-Lagrange strain error as a function of normalized longitudinal position (b-d). Increasing amount of shift leads to an increased error in displacement field (a). Radial strain error increases towards the base and apex, especially for the maximum amount of shift, ±6 mm, while longitudinal and circumferential components are insensitive to any shift (d).

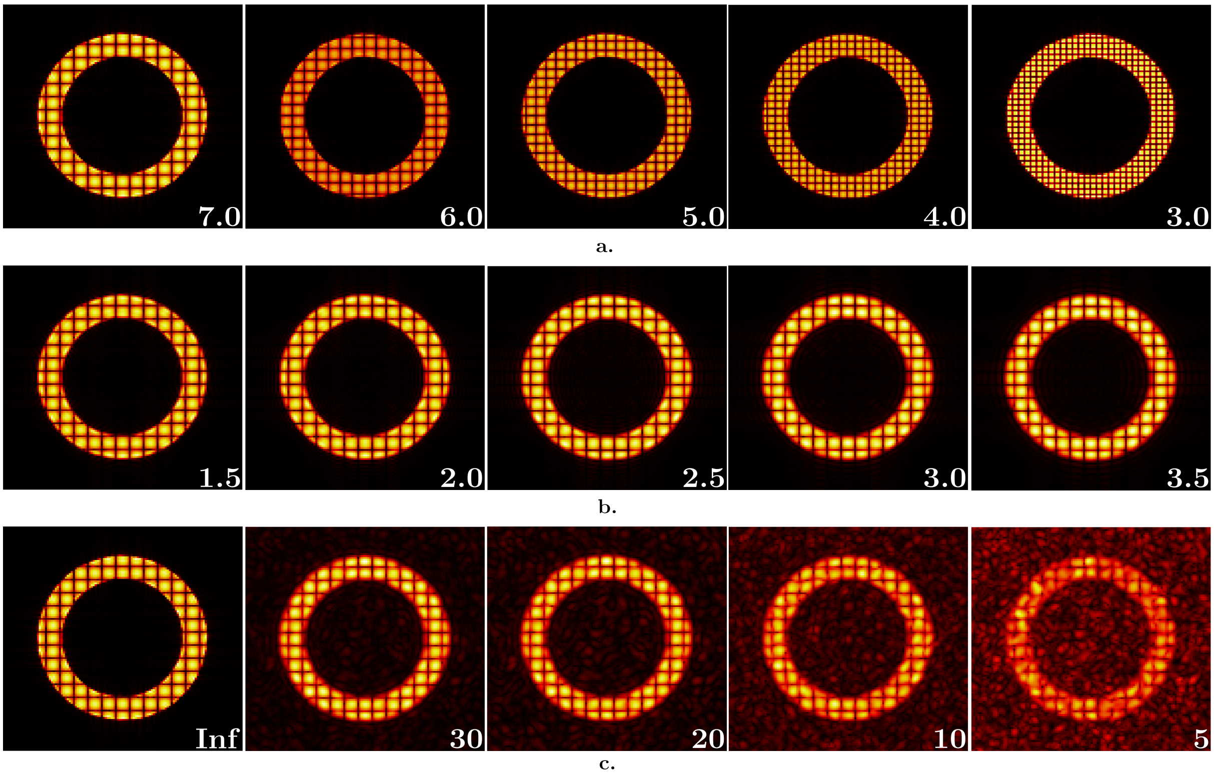

Fig 2. Isotropic synthetic images to analyze the effect of image properties. Images with varying tag distances (in mm) (a), image resolutions (in mm) (b), and SNRs (c), decreasing from left to right (values are written at the bottom right corner for each case). Images shown in (a) are noiseless and have 1 mm spatial resolution. Images in (b) are noiseless and have 7 mm tag distance. For the ones in (c), image resolution and tag distance are 3.5 mm and 7 mm, respectively. Images represent the configuration at end-diastolic time frame.

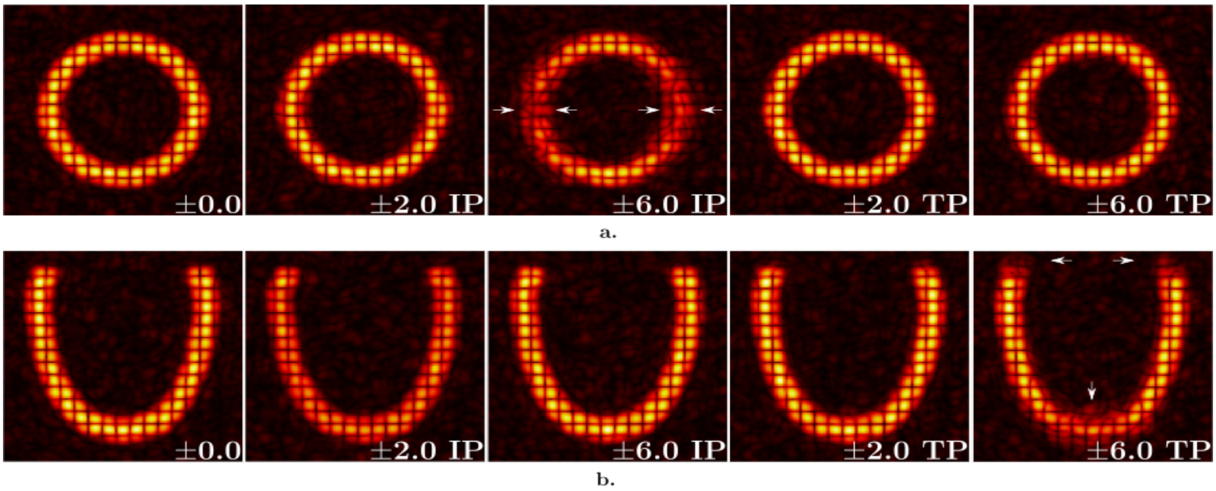

Fig 3. Anisotropically resolved synthetic images to analyze the effect of geometrical inconsistencies. Images with 7 mm tag distance, SNR 20, and 3.5x7.0x7.0 mm3 resolution for varying amounts of shift increasing from left to right (shift magnitude in mm are written at the bottom right corner for each case) on short-axis (a) and long-axis (b) views. IP and TP stand for the direction of shift, in-plane and through-plane, respectively. Images represent the configuration at end-diastolic time frame. The arrows indicate the regions with the largest amount of error due to shift for IP (±6 IP) and TP (±6 TP).

References

[1] M. Genet, C. T. Stoeck, C. Von Deuster, L. C. Lee, and S. Kozerke, “Equilibrated Warping: Finite Element Image Registration with Finite Strain Equilibrium Gap Regularization,” *Med. Image Anal.*, vol. 50, pp. 1–22, 2018.

[2] A. K. Rutz, S. Ryf, S. Plein, P. Boesiger, and S. Kozerke, “Accelerated whole-heart 3D CSPAMM for myocardial motion quantification,” *Magn. Reson. Med.*, 2008.
