# Supplementary material for: In-silico study of accuracy and precision of left-ventricular strain quantification from 3D tagged MRI
Source: PLoS One. 2021 Nov 5;16(11):e0258965. doi: 10.1371/journal.pone.0258965 (PMC8570486; doi:10.1371/journal.pone.0258965)
Supplement: Attachment — Submitted filename: Response to Reviewers.pdf [file pone.0258965.s003.pdf]

We would like to thank the reviewer for carefully reading our revised manuscript, and making constructive comments. We have addressed all the comments in the following point by point response:

Reviewer #2

The manuscript has been skillfully revised by the authors.

Thank you very much.

After examining the revised manuscript, I still have two queries for the authors, but these should only require very minor changes to the manuscript.

We have improved the revised manuscript considering the reviewer's comments carefully.

1) Equation 1 implies a very simply preparation for spatial modulation of magnetization, while in practice e.g. a binomial scheme is going to result in sharper tag lines. The authors could point out that in this sense there are potentially looking at a worst case scenario in terms of tag line contrast.

We thank the reviewer for this comment. As it is indicated, here we assume a basic image generating function used in standard CSPAMM, which implements a low order of 1-1 binomial pattern. Thereby, we have less tag line contrast compared to other tagging techniques, e.g. SPAMM, in which the binomial tagging pattern is approximated with higher degrees of functions. Referring to [1], we clarified this very relevant point in Section 1.2 (please note that this citation did not cause any change in Section "References"):

"We assume a standard image generating function used in CSPAMM to create the tagged image stacks:

$$I = \sin\left(\frac{\pi X_i}{s}\right), \quad (1)$$

where X_i corresponds to the spatial coordinate of the image pixel in the ith direction, and s is the tagging distance, ranging from 3 mm to 7 mm (Fig 2.a). Compared to other tagging techniques, in which the tagging pattern is approximated by higher order functions, (e.g., SPAMM) [1], here we assume the standard modulation pattern for CSPAMM as a "worst case" scenario in terms of tag line contrast."

2) The authors state in the legend for the new figure S1: "The tracking algorithm requires a higher regularization strength as image resolution and SNR decrease." Could the authors clarify if image resolution causes an increase of the regularization weight independent of the SNR, or if the effect of image resolution is through the natural decrease of SNR?

We thank the reviewer for clarifying this point. As it is illustrated in S1 Fig, we have two varying image properties; image resolution and SNR that determine the regularization strength. Keeping SNR constant, decreasing pixel size requires less regularization. Likewise, if there is no change in pixel size, a decrease in SNR is compensated with higher regularization.

We restated that point in Section “Supporting Information”:
“For isotropic image resolution (S1 Fig.a), change in SNR does not affect the value of optimal regularization strength for small pixel size (1 mm). However, for larger pixel size, regularization strength increases with decreasing SNR. Similarly, for a given SNR, increasing pixel size is compensated with higher regularization strength. For the worst case (pixel size = 3.5 mm and SNR 5), the image registration requires β= 0.2. For anisotropic image resolution (S1 Fig.b), the dependency of regularization strength on SNR and pixel size follows the same trend as in isotropic images. For the worst case (pixel size = 3.5x7.0x7.0 mm^3 and SNR 5), β= 0.3 is required.”

and also, S1 Fig’s caption:

“S1 Fig. Change in optimal regularization strength with respect to image resolution and SNR. Colour code represents the value of regularization strength. The pixel size (in mm) in one direction is shown on x axis for isotropic images (a). SNR has more impact on the choice of regularization strength as pixel size increases. For anisotropic images, 3D image resolution is represented on x axis (in mm^3) (b). For a given SNR, increasing pixel size requires more regularization. Likewise, higher regularization is required for decreasing SNR when pixel size is kept constant.”

It would make sense that higher image resolution does require stronger regularization, as the higher image resolution is likely increasing how ill-posed the inverse problem is.

We thank the reviewer for this comment. In our image registration technique, we expect a decrease in regularization strength as image pixel size gets smaller. By implementation, the number of degrees of freedom in displacement field is independent from the image discretization as our displacement field is defined on the mesh. Moreover, the smaller the pixel size, less partial voluming we have, resulting in a more accurate description of the displacement. Hence, regularization has less to accommodate for that.

I am also not sure if what they call a "decrease" of image resolution is correct terminology, because if pixel size decreases I would call this "higher resolution" and not a decrease of resolution. The authors should consult with a native speaker.

We apologize for this misunderstanding and agree with the reviewer that decrease in pixel size means “higher resolution”. We checked the manuscript carefully to find all appearances of “resolution”, listed below in bold. We have replaced it with “pixel size” if it refers to a quantity, or we mention about a decrease/increase, and showed these changes in red.

in Section “Abstract”:

“To this end, synthetic biomechanical left-ventricular tagged MRI data with varying tag distance, **spatial resolution** and signal-to-noise ratio (SNR) were generated and processed to quantify errors in radial, circumferential and longitudinal strains relative to ground truth.”

“Results reveal that radial strain is more sensitive to **image resolution** and noise than the other strain components.”

“In conclusion, our study points to the need for **higher-resolution** 3D tagged MRI than currently available in practice in order to achieve sufficient accuracy of radial strain quantification.”
in Section “Introduction”:

“Limitations include a reduction of **spatial resolution** due to band-pass filtering [22], as well as potential phase aliasing [23].”

“It is the objective of the present work to analyze the dependency of radial, circumferential and longitudinal strain quantification from 3D tagged MRI with regard to tag distance**, image resolution** and SNR.”

in Section 1:

“Fig 1. Study design. Reference biomechanical LV model simulating the full cardiac cycle (a). Synthetic image generation using the model (b). Three different image stacks with tag lines in orthogonal directions are generated by rasterization (1) and then resampled by cropping in k-space and convolved in time (2). Adding noise and changing **image resolution** by filtering in k-space (c). Image registration showing 3D tagged images superimposed with the warped mesh for different time frames (d).”

in Section 1.2:

“First, tagged images with 0.5 mm **pixel size** are generated from the time-resolved LV meshes through rasterization [39], and using a simplified complementary spatial modulation model [6] for 3D tagging without tag-line fading (Fig1.b-1).”

“Thereafter, images are resampled to have 1 mm **pixel size** using zero-filling after cropping in k-space and convolution in time (Fig 1.b-2) using a convolution window size of 40 ms [9].”

“Then, image **spatial resolution** is changed by filtering the reference dataset in k-space using a box filter with different bandwidth (Fig 1.c). We generated image sets varying in tag distance (Fig 2.a), **pixel size** (Figs 2.b), and SNR (Fig 2.c).”

“Fig 2. Isotropic synthetic images to analyze the effect of image properties. Images with varying tag distances (in mm) (a), **pixel sizes** (in mm) (b), and SNRs (c) (values are written at the bottom right corner foreach case). Images shown in (a) are noiseless and have 1 mm **pixel size**. Images in (b) are noiseless and have 7 mm tag distance. For the ones in (c), **pixel size** and tag distance are 3.5 mm and 7 mm, respectively. Images represent the configuration at end-diastolic time frame.”

in Section 1.4:

“In this study, we investigate both the individual and combined effects of two image characteristics, Tag distance to Pixel size Ratio (TPR) and SNR on images with isotropic (Fig 2) and anisotropic (Fig 3) **spatial resolution**. The first part, analysis on isotropic images, includes noiseless images with TPR ranging from 2.8 to 7.0 (Fig2. (a,b)), and images with varying levels of SNR (from 5 to 30) (Fig 2.c), keeping the tag distance and **pixel size** constant: 7 mm and 1 mm, respectively. The combined effect of TPR and SNR is investigated on the

image set with isotropic **pixel size** ranging from 1.5 mm to 3.5 mm having different SNRs (from 5 to 30) and a tag distance of 7 mm."

"Fig 3. Anisotropically resolved synthetic images to analyze the effect of geometrical inconsistencies. Images with 7 mm tag distance, SNR 20, and 3.5x7.0x7.0 mm^3 **pixel size** for varying amounts of shift increasing from left to right (values are written at the bottom right corner for each case) on short-axis (a) and long-axis (b) views. IP and TP stand for the direction of shift, in-plane and through-plane, respectively. Images represent the configuration at end-diastolic time frame. The arrows indicate the regions with the largest amount of error due to shift for IP (±6 IP) and TP (±6 TP)."

"The second part is focused on the analysis of anisotropic **image resolutions**. Similarly, individual effects of **resolution** and SNR are analysed first. For this purpose, we generated noiseless images with 7 mm tag distance and **pixel size** ranging from 1.5 mm to 3.5 mm in the tagging direction, while the transverse directions are assigned to a **larger pixel size** ranging from 3.5 mm to 7 mm.
For the SNR analysis, three datasets are chosen to represent different levels of **pixel size** (2.0x4.0x4.0 mm^3, 3.0x6.0x6.0 mm^3, and 3.5x7.0x7.0 mm^3) with varying noise levels in the same interval as used for the isotropic case."

"In this study, we simulated in-plane (IP) and through-plane (TP) shifts of image stacks with 3.5x7.0x7.0 mm^3 **pixel size** and SNR 20.

<u>in Section 2.1.1:</u>

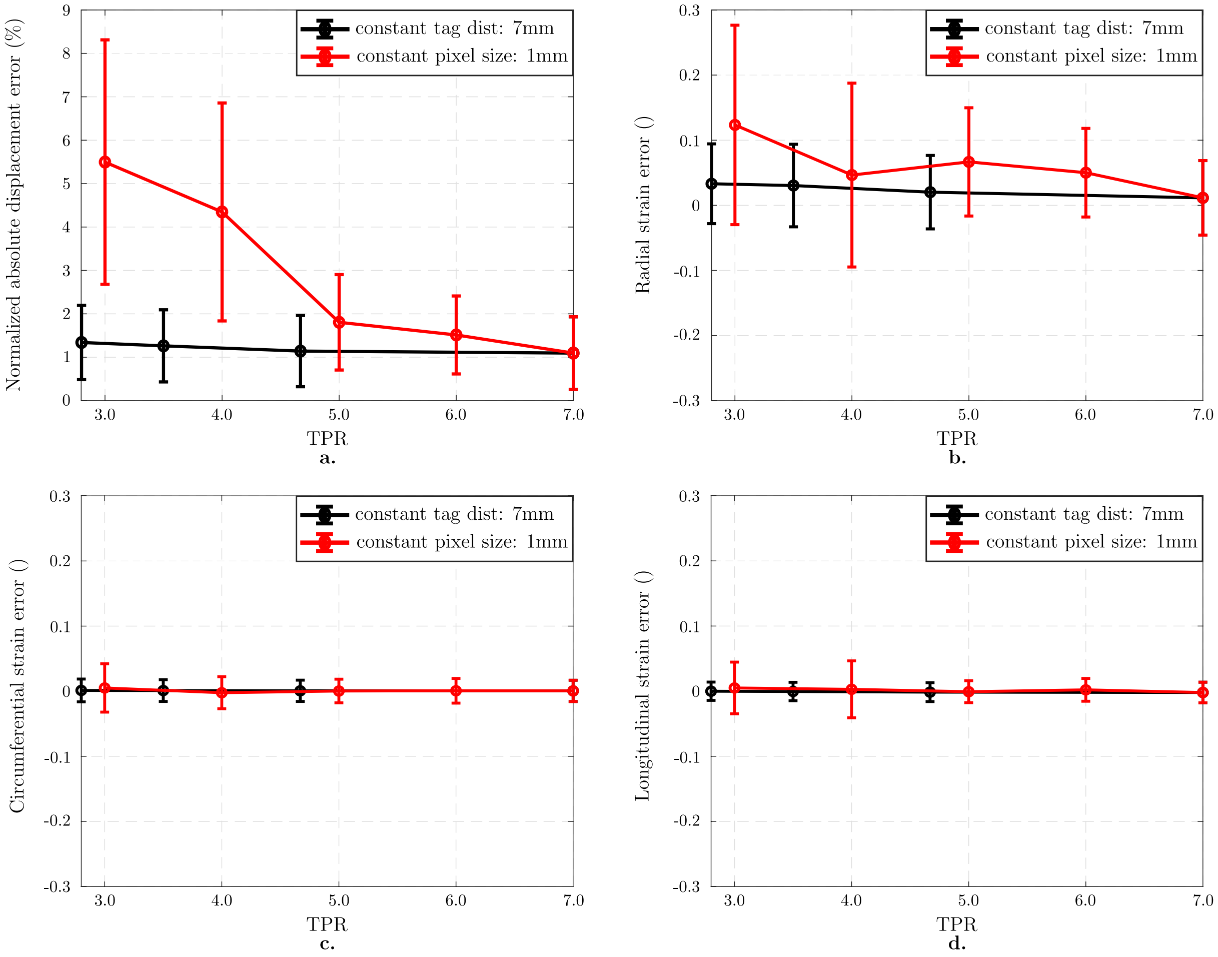

Fig 4. Effect of Tag distance to Pixel size Ratio (TPR) on noiseless isotropic image analysis. Normalized mean ± standard deviations in displacement error norm (%) plotted as a function of TPR (a). Black curve stands for the errors computed for the images with different **pixel sizes** keeping the tag distance constant at 7 mm while the red one represents the analysis results with different tag distances where the **pixel size** is 1 mm. Mean ± standard deviations in component-wise Green-Lagrange strain error as a function of TPR for the best performing regularization strength (b-d). Radial strain component (b) is more sensitive to a change in TPR while the circumferential (c) and the longitudinal (d) components are more accurately measured.

"Fig 4 presents the error analysis as a function of TPR, where the black curves represent change in **pixel size** for a constant tag distance of 7 mm while the red curves stand for constant **pixel size** of 1 mm as tag distance varies."

"The largest strain error is observed in the radial component, which is 0.1 ± 0.2 for 1 mm **pixel size** and 3 mm tag distance (Fig 4.b)."

<u>in Section 2.1.2:</u>

"Fig 5. Effect of SNR on isotropic image analysis. Results on images with 1 mm **pixel size** and 7 mm tag distance. Normalized mean ± standard deviations in displacement error norm (%) without regularization (red curve) and with optimal regularization (black curve) (a). Regularization helps decreasing the registration error significantly. Mean ± standard deviations in component-wise Green-Lagrange strain error as a function of SNR which is independent of regularization strength for SNR≥20(b-d)."

<u>in Section 2.1.3:</u>

"Fig 6 presents the results for the images with different isotropic **resolutions** varying in SNR at a fixed tag distance of 7 mm. Error in displacement field significantly increases as **pixel size** and SNR **change** from 1.0 mm to 3.5 mm and Inf to 5, respectively (Fig 6.a). While the error in displacement field is 1.1 ± 0.8 % for 1 mm **pixel size** and Inf SNR, it increases to 3.1 ± 1.8 % when **pixel size** is 3.5 mm and SNR 5 (worst case)."

"The reader is referred to S1 Fig.a for the choice of optimal β values for varying **image resolution** and SNR. The two-way ANOVA analysis on SNR and **image resolution** shows that SNR is the main source of error in displacement field, radial and circumferential strain errors with 56%, 69% and 39%, respectively, while the corresponding contributions from **image resolution** are 39%, 20% and 37%. The longitudinal strain error, however, is 17% due to SNR and 72% due to **image resolution** ($p<0.0001$)."

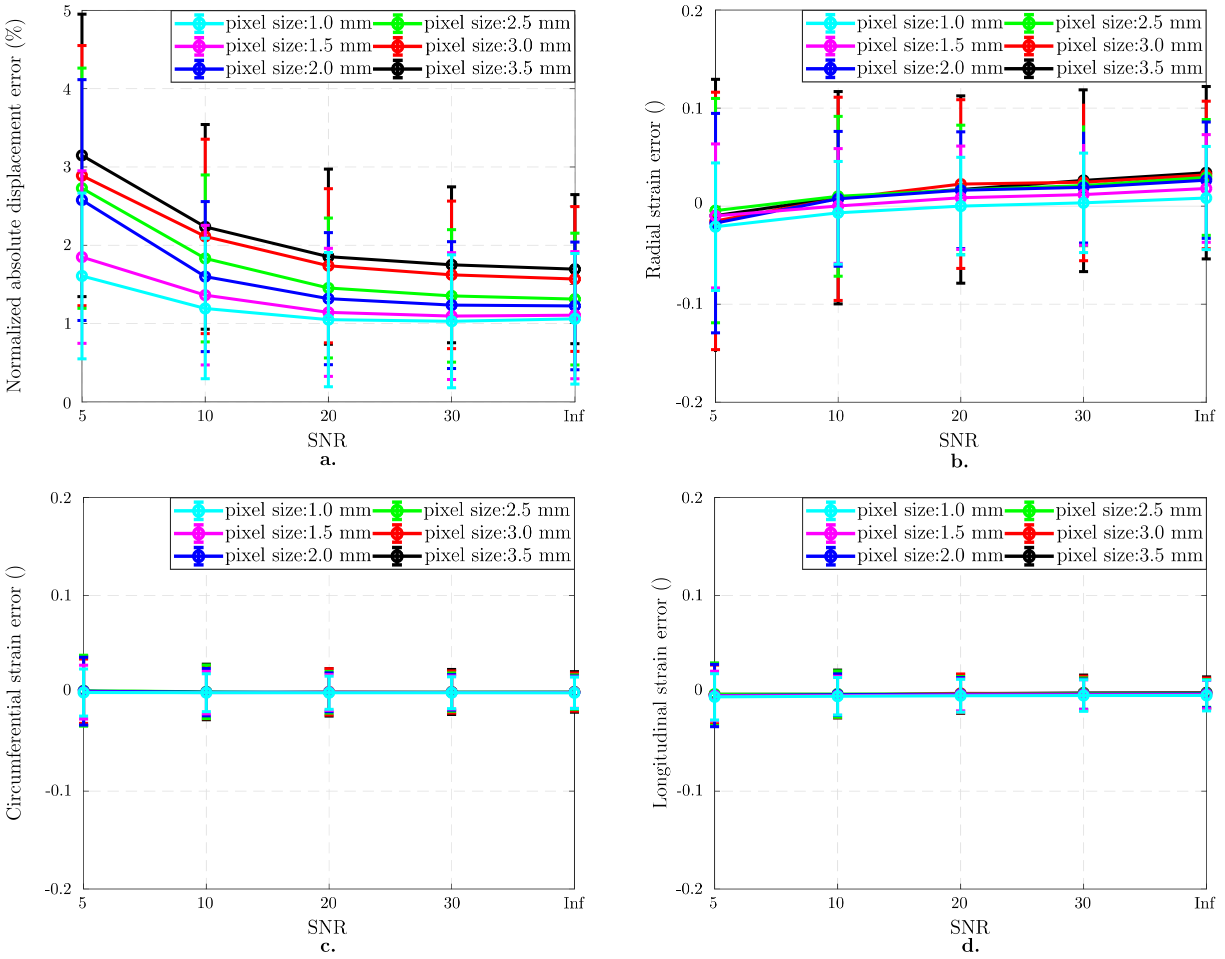

Fig 6. Combined effect of **image resolution** and SNR on isotropic image analysis. Results on images with 7 mm tag distance. Normalized mean ± standard deviations in displacement error norm (%) plotted as a function of SNR for different **pixel sizes** (a). Mean ± standard deviations in component-wise Green-Lagrange strain error as a function of SNR and **pixel size** (b-d). Combined effect of **pixel size** and SNR is more pronounced on the radial strain component (b) while there is almost no change in the circumferential (c) and longitudinal (d) components. Legends show the **pixel size** in mm.

<u>in Section 2.2.1:</u>

"2.2.1 Impact of **image resolution**"

"For each plot, the x axis represents the **pixel size** in tagging direction which is finer compared to the orthogonal (transverse) directions represented by the y axis. Fig 7.a shows the normalized mean ± standard deviation (%) of the displacement error norm which increases from 1.5 % to 2.6 % as the **pixel size increases** from 1.5x3.5x3.5 mm^3 to 3.5x7.0x7.0 mm^3. The error in radial strain increases up to 0.06 when **the pixel size in orthogonal direction is larger than** 5 mm (Fig 7.b). Moreover, circumferential (Fig 7.c) and longitudinal (Fig 7.d) components are less sensitive to change in **image resolution**."

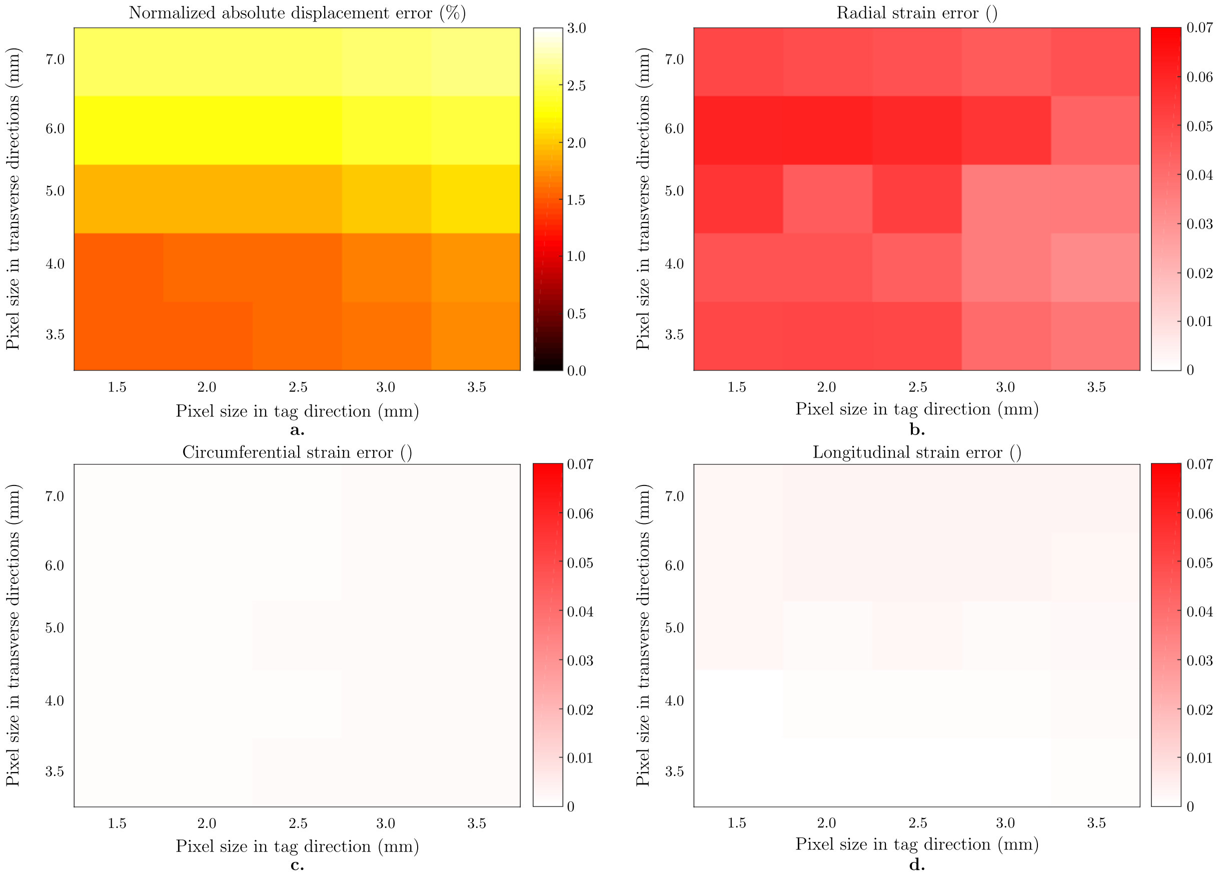

Fig 7. Effect of **image resolution** on anisotropically resolved image analysis. Results on noiseless images with 7 mm tag distance. Normalized mean ± standard deviations in displacement error norm (%) as a function of **pixel size** (a). Signed averages in Green-Lagrange strain component errors as a function of **pixel size** (b-d). Radial strain component is the most sensitive to an **increase in pixel size** (b), while circumferential (c) and the longitudinal (d) components are not affected at all.

in Section 2.2.2:

"2.2.2 Combined impact of **image resolution** and SNR"

"We observe an increase in error both in displacement field (Fig 8.a) and strain components (Fig 8.(b-d)) for anisotropic images as SNR **decreases and pixel size increases**. The lowest error was found for the **smallest pixel size** and highest SNR: the error in displacement field is 1.5 ± 1.0 % while it increases to 4.4 ± 2.8 % for the worst case (3.5x7.0x7.0 mm^3 **pixel size** & SNR 5) (Fig 8.a). The reader is referred to S1 Fig.b for the choice of optimal β values for **anisotropic image resolutions** (2.0x4.0x4.0 mm^3, 3.0x6.0x6.0 mm^3 and 3.5x7.0x7.0 mm^3) and varying SNR. ANOVA analysis shows that SNR is the main source of error in displacement and radial, circumferential and longitudinal strain errors with values of 62%, 78%, 64%, and 46%, respectively while **image resolution** contributes with 36%, 1%, 16%, and 24% to each field (for $p<0.0001$)."

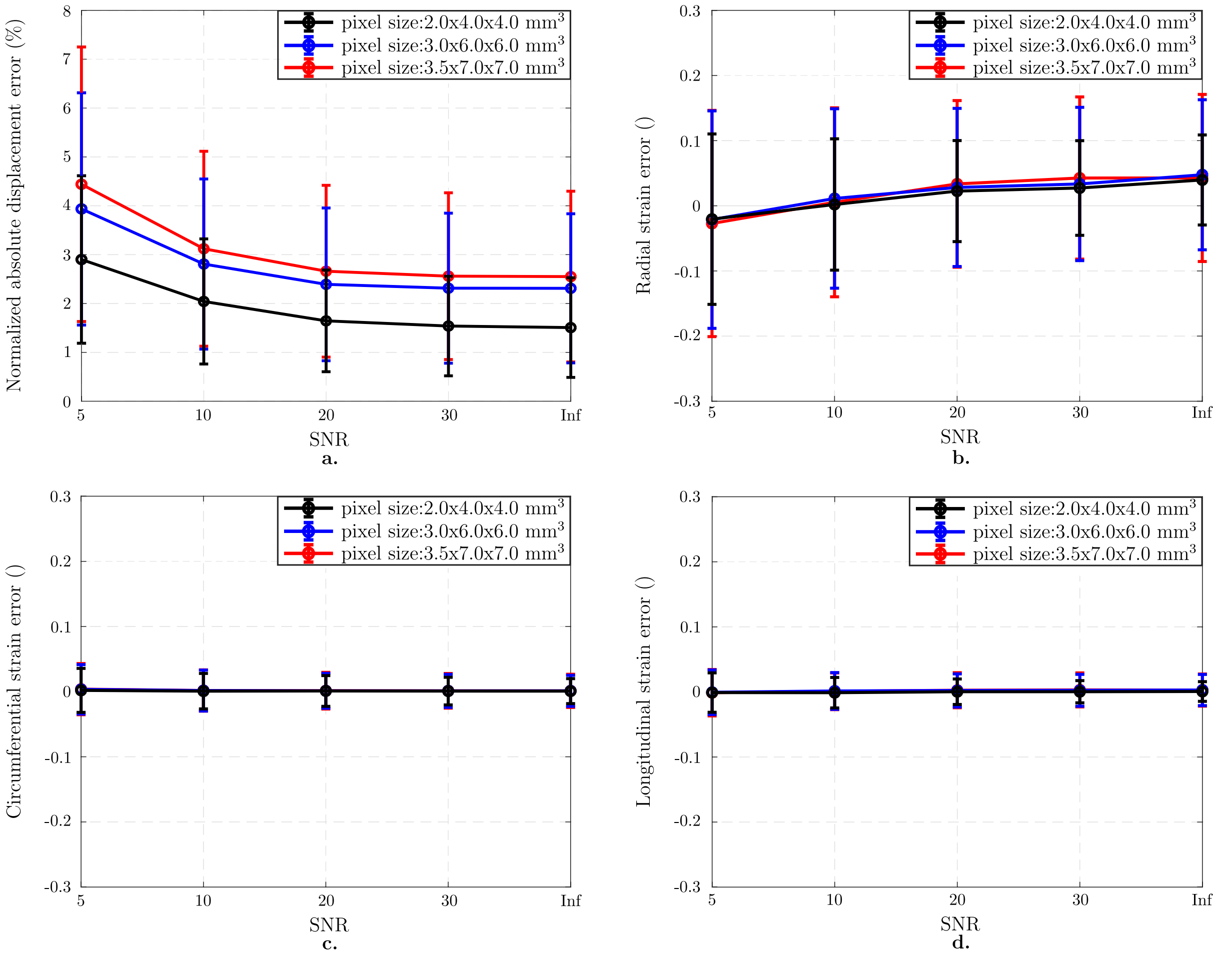

Fig 8. Combined effect of **image resolution** and SNR on anisotropically resolved image analysis. Results on images with 7 mm tag distance. Normalized mean ± standard deviations in displacement error norm (%) plotted as a function of SNR for different **pixel sizes** (a). Mean ± standard deviations in component-wise Green-Lagrange strain error as a function of SNR and **pixel size** (b-d). Combined effect of **increased pixel size and decreased** SNR is more pronounced on the radial strain component (b) while there is almost no change in the circumferential (c) and longitudinal (d) components.

<u>in Section 2.2.3:</u>

"2.2.3 Combined impact of **image resolution**, SNR and geometrical inconsistencies"

"Fig 9 presents the results for the images with anisotropic **resolution** 3.5x7.0x7.0 mm^3, tag distance of 7 mm and SNR 20 when different amounts of shift between tagged image stacks are applied."

"Fig 9. Effect of geometrical inconsistencies. Results on images with anisotropic **pixel size** 3.5x7.0x7.0 mm^3 and SNR 20 for 7 mm tag distance. Normalized mean ± standard deviations in displacement error norm (%) plotted as a function of in-plane and through plane shift (in mm) between image stacks (a). Mean ± standard deviations in component-wise Green-Lagrange strain error as a function of in-plane and through plane shift (in mm) between image stacks (b-d). Increasing amount of shift leads to an increased error in radial strain component (b) while there is almost no change in the circumferential (c) and longitudinal (d) components."

in Section “Discussion”:

“We have investigated the effect of image **resolution**, tag distance and SNR on deformation quantification from 3D tagged images and have illustrated the challenges in quantifying radial strain.”

“Although TPR is a dimensionless parameter essentially controlling the impact of tag distance and **image resolution** on the motion tracking, the tracking error still differs when varying TPR by changing tag distance (red curve of Fig 4) or pixel size (black curve of Fig 4).”

“Focusing on results with 7 mm tag distances (black curve of Fig 4), tracking results are not drastically influenced by TPR, i.e., by **image resolution**.”

“Radial strain error is generally larger than circumferential and longitudinal strain errors; however, this higher sensitivity of radial strain is not significantly affected by **image resolution**.”

“Looking at the combined effect of **image resolution** and SNR (see Fig 6), we observed a considerable increase in displacement error with **increasing pixel size and decreasing** SNR. This trend is seen despite the use of optimal regularization and fine temporal discretization, which suggests that the cause may be found in the image properties, i.e., **spatial resolution** and SNR.”

“Alternatively, for future CMR acquisitions, tagged **image resolution** needs to be improved for better radial strain mapping.”

“And again, since this is found with a fine temporal discretization and an optimal motion tracking method, this reinforces the point that reduced radial strain measured on in vivo images is induced by limited image **spatial resolution** and SNR.”

“Nevertheless, our study suggests that by improving **image resolution**, measuring accurate radial strain fields could be achieved based solely on tagged MRI with existing image processing techniques.”

“Moreover, the error analyses suggest that it is worthwhile investing into **higher spatial resolution** when planning tagged CMR acquisitions in order to obtain a robust radial strain estimate.”

in Section “Supporting Information”:

“S1 Fig represents the dependency of the optimal regularization parameter on **image resolution** and SNR.”

“For isotropic **image resolution** (S1 Fig.a), change in SNR does not affect the value of optimal regularization strength for small **pixel size** (1 mm). However, for larger **pixel size**, regularization strength increases with decreasing SNR. Similarly, for a given SNR, increasing **pixel size** is compensated with higher regularization strength. For the worst case (pixel size = 3.5 mm and SNR 5), the image registration requires β= 0.2. For anisotropic **image resolution** (S1 Fig.b), the dependency of regularization strength on SNR and **pixel** size follows the same

trend as in isotropic images. For the worst case (**pixel size** = 3.5x7.0x7.0 mm^3 and SNR 5), β= 0.3 is required."

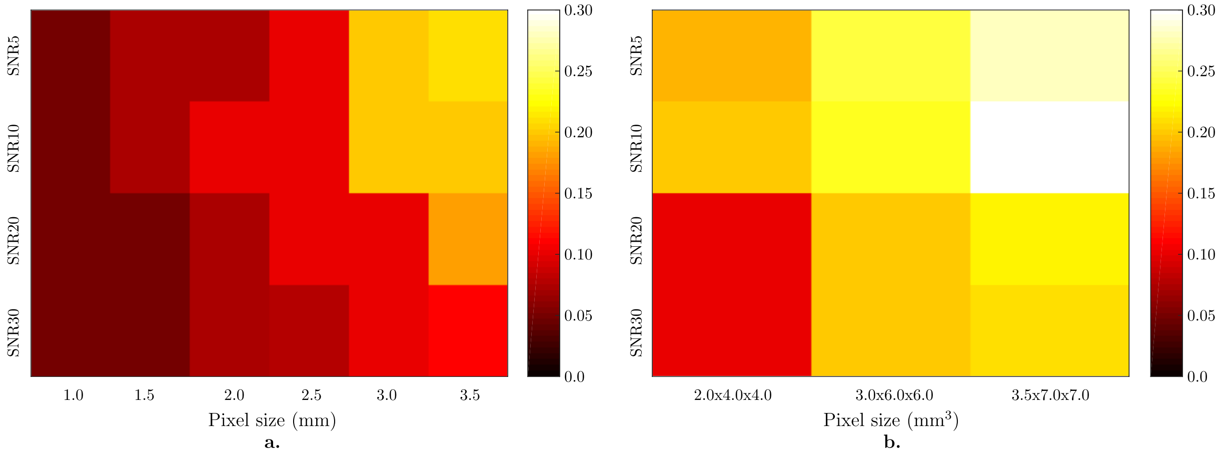

S1 Fig. Change in optimal regularization strength with respect to **image resolution** and SNR. Colour code represents the value of regularization strength. The **pixel size** (in mm) in one direction is shown on x axis for isotropic images (a). SNR has more impact on the choice of regularization strength as **pixel size increases**. For anisotropic images, 3D **image resolution** is represented on x axis (in mm^3) (b). For a given SNR, increasing **pixel size** requires more regularization. Likewise, higher regularization is required for decreasing SNR when **pixel size** is kept constant.

"S2 Fig. Effect of in-plane shift on regional motion quantification. Results on images with anisotropic **pixel size** 3.5x7.0x7.0 mm^3 and SNR 20 for 7 mm tag distance. For all the plots, colour code shows the amount of in-plane shift ranging from ±0 mm to ±6 mm. Mean ± standard deviation in normalized displacement error norm (%) plotted as a function of in-plane angle, α with respect to shift direction (a). Mean ± standard deviations in component-wise Green-Lagrange strain error as a function of in-plane angle (b-d). Increasing amount of shift leads to an increased error in displacement field (a). Radial and circumferential strain errors show the dependency on in-plane angle, especially for the maximum amount of shift, ± 6 mm. The shifts are applied at $\alpha = 0^\circ$ and $\alpha = 180^\circ$, shown with the green arrows on top (b, c). Longitudinal component is insensitive to any shift (d)."

"S3 Fig. Effect of through-plane shift on regional motion quantification. Results on images with anisotropic **pixel size** 3.5x7.0x7.0 mm^3 and SNR 20 for 7 mm tag distance. For all the plots, colour code shows the amount through-plane shift ranging from ±0 mm to ±6 mm. Mean ± standard deviation in normalized displacement error (%) plotted as a function of normalized longitudinal position (a). Mean ± standard deviations in component-wise Green-Lagrange strain error as a function of normalized longitudinal position (b-d). Increasing amount of shift leads to an increased error in displacement field (a). Radial strain error increases towards the base and apex, especially for the maximum amount of shift, ±6 mm, while longitudinal and circumferential components are insensitive to any shift (d)."

3) Additionally, we found typos in the text which were corrected as follows:

<u>in Section 1.2, Fig 2's caption (changing end-systolic to end-diastolic):</u>

"Images represent the configuration at end-diastolic time frame."

in Section 1.3 (reformatting the sentence):

"The image similarity metric, Ψ^{im},

$$\Psi^{\mathrm{im}}(\mathbf{U}) = \frac{1}{2}\|I_t \circ \mathbf{\Phi} - I_0\|^2_{L^2(\Omega_0)}, \tag{5}$$

and the regularization energy, Ψ^{reg},

$$\Psi^{\mathrm{reg}} = \sum_K \frac{1}{2}\|\mathbf{Div}(\mathbf{F}.\mathbf{S})\|^2_{L^2(K)} + \sum_F \frac{1}{2h}[\![\mathbf{F}.\mathbf{S}.\mathbf{N}]\!]^2_{L^2(F)} \tag{6}$$

are weighted by factors (1- β) and β, respectively, to rescale the regularization strength to stay in the range $[0,1]$. K, F and **N** represent the set of finite elements, the set of interior facets, and the facets normal, respectively."

in Section 1.4 (removing the bars from $\bar{e}_{avg}$ and $\bar{e}_{std}$):

"In addition to the displacement error, we investigate the component-wise sensitivity of the strain to a change in image characteristics by computing the mean and standard deviation according to:

$$\mathrm{e_i} = \mathrm{s^i_{reg}} - \mathrm{s^i_{ref}} \tag{11}$$

$$\mathrm{e_{avg}} = \frac{1}{\mathrm{N_{el}}}\sum_{\mathrm{i=1}}^{\mathrm{N_{el}}} \mathrm{e_i} \tag{12}$$

$$\mathrm{e_{std}} = \frac{1}{\mathrm{N_{el}}}\sum_{\mathrm{i=1}}^{\mathrm{N_{el}}} \left(\mathrm{e_i} - \mathrm{e_{avg}}\right)^2 \tag{13}$$

where $\mathrm{e_{avg}}$ and $\mathrm{e_{std}}$ are the mean and standard deviation in strain error for components, which is a scalar field, while $\mathrm{s^i_{reg}}$ and $\mathrm{s^i_{ref}}$ are the strain values at element i at end-systolic time frame for the registered case and the ground truth, respectively."

Fig 3's caption (changing end-systolic to end-diastolic, and adding the second sentence):

"Images represent the configuration at end-diastolic time frame. The arrows indicate the regions with the largest amount of error due to shift for IP (±6 IP) and TP (±6 TP)."

in Section 2.2.3 (removing the white space after "±"):

"When the image stacks are shifted by ±6 mm in-plane and through plane, the error increases up to 10.1 ± 5.4 % and 9.8 ± 5.0 %, respectively."

"The error is -0.02 ± 0.27 when the shift is ±6 mm in-plane while it is -0.01 ± 0.32 for through plane shift."

in Section "Supporting information" (removing the white space after "±"):

"For both the displacement (S2 Fig.a) and strain components (S2Fig.b, c), maximum errors are observed for the largest amount of shift, ±6 mm (shown by the red curves)."

"For the maximum amount of shift (±6 mm), the displacement and radial strain error get larger towards the apex and base compared to lower amounts of shift."

S2 Fig's caption (removing the white space after "±"):

"For all the plots, colour code shows the amount of in-plane shift ranging from ±0 mm to ±6 mm."

"Radial and circumferential strain errors show the dependency on in-plane angle, especially for the maximum amount of shift, ±6 mm."

S3 Fig's caption (removing the white space after "±"):

"For all the plots, colour code shows the amount through-plane shift ranging from ±0 mm to ±6 mm."

"Radial strain error increases towards the base and apex, especially for the maximum amount of shift, ±6 mm, while longitudinal and circumferential components are insensitive to any shift (d)."

References

[1] S. E. Fischer, G. C. McKinnon, S. E. Maier, and P. Boesiger, "Improved myocardial tagging contrast," *Magn. Reson. Med.*, 1993.
